# The *Ggcx^K325Q^* Mutation Does Not Affect the Calcium Homeostasis of the Epididymis and Male Fertility in Mice

**DOI:** 10.3390/cimb46060303

**Published:** 2024-05-22

**Authors:** Mingxiang Xiong, Pang Cheng, Bo Liu, Yanqiu Zhao, Ting Gao, Zhen Li

**Affiliations:** 1 College of Life Sciences, Northwest University, Xi’an 710069, China; xiongmingxiang@stumail.nwu.edu.cn; 2 Department of Human Anatomy, Histology and Embryology, Air Force Medical University, Xi’an 710038, China; chengpang0524@163.com (P.C.); histolbo@163.com (B.L.); 15865313882@163.com (Y.Z.); gt16673408056@126.com (T.G.)

**Keywords:** rs699664, GGCX, MGP, calcium homeostasis, epididymis

## Abstract

A low-calcium microenvironment is imperative for spermatozoa maturation within the epididymis. Our previous work has shown that γ-glutamyl carboxylase (GGCX), the carboxylation enzyme of the matrix Gla protein (MGP), plays an essential role in epididymal calcium homeostasis and sperm maturation in rats and that the GGCX SNP mutation rs699664 was associated with asthenozoospermia (AZS) in humans. Here, we investigated the expression patterns of GGCX and MGP in the mouse epididymis and generated *Ggcx^K325Q^* knock-in (KI) mice. We also tested the effects of this mutation on epididymal calcium homeostasis, sperm function, and male fertility in *Ggcx^K325Q−/−^* mice. The results showed that both GGCX and MGP were enriched in all regions of the mouse epididymis, especially in the initial segment of the epididymis. Double immunofluorescence staining revealed that GGCX colocalized with MGP in the epithelial cells of the initial segment and caput regions as well as in the lumen of the corpus and cauda regions of the mouse epididymis. However, the *Ggcx^K325Q−/−^* mice were fertile with normal epididymal morphology, sperm functions, and epididymal calcium concentration. Overall, our findings revealed that the *Ggcx^K325Q^* mutation does not exert any discernible effect on male fertility in mice.

## 1. Introduction

The mammalian epididymis is a long, convoluted tubule that plays an important role in sperm transport, concentration, protection, and storage [1]. Generally, the mouse epididymis is divided into four main anatomical regions: initial segment, caput, corpus, and cauda. Each epididymal region possesses regionalized and fine-tuned gene expression patterns that are related to physiological functions important for sequential steps in sperm maturation [2]. A low calcium concentration is a unique feature of the epididymal luminal microenvironment [3]. An abnormally high epididymal Ca^2+^ concentration can lead to impaired sperm motility, reduced viability, and compromised fertilization capacity, resulting in male infertility [4]. Therefore, the homeostasis of epididymal calcium is essential for normal male fertility [5,6,7]. Multiple pathways have been found to regulate epididymal Ca^2+^ homeostasis, such as the vitamin D-related TRPV6-TMEP16A pathway and the vitamin K-dependent gamma-glutamyl carboxylase (GGCX)-matrix Gla protein (MGP) pathway [8]. However, the mechanisms underlying the regulation of calcium homeostasis in the epididymis are not fully understood.

The GGCX-MGP system has been reported to play a major role in maintaining the Ca^2+^ balance in different tissues, such as the vasculature and cartilage tissue [9,10]. As the only vitamin K-dependent carboxylase in mammals, GGCX is widely distributed in many organs and tissues, such as the liver, kidney, testis, and epididymis [11,12,13]. It relies on vitamin K2 to catalyze dehydrogenation and carboxylation of the γ-carbon atom of glutamic acid residues (Glu) in the substrate protein, resulting in the formation of γ-carboxylated glutamate (Gla) [14,15]. This post-translational modification is essential to several coagulation factors, such as prothrombin, factor VII, factor IX, protein C, protein S, and protein Z [16]. Defects in the γ-carboxylation of these coagulation factors will cause bleeding disorders, referred to as combined vitamin K-dependent coagulation factors deficiency (VKCFD) [17]. Other notable proteins targeted by γ-carboxylation include osteocalcin and MGP [18,19,20]. MGP is a secreted calcium-binding protein involved in inhibiting calcification in several tissues, such as vasculature, cartilage tissue, and dermal fibroblasts [21,22,23]. Carboxylated MGP is a potent inhibitor of connective tissue mineralization and vascular calcification. It can regulate the Ca^2+^ concentration by binding free calcium ions and calcic crystals [24,25]. Defects in MGP carboxylation have been linked to a variety of diseases, including cardiovascular diseases, pseudoxanthoma elasticum-like syndrome, and Keutel syndrome [10,26,27]. MGP knockout mice die of vessel ruptures within two months of birth due to massive vascular calcification, indicating that MGP is indispensable during development [9]. Our previous study found that both GGCX and MGP were expressed in the rat epididymis and confirmed that the GGCX-MGP system plays an essential role in maintaining the low calcium environment of the epididymal lumen. When the carboxylation of MGP was inhibited, the calcium homeostasis in the rat epididymis was dysregulated, leading to male infertility [12].

Our previous study identified an SNP, rs699664, in the *GGCX* gene of infertile men diagnosed with asthenozoospermia (AZS) [12]. However, whether the rs699664 mutation of GGCX could affect the calcium homeostasis of epididymis in humans and further affect male fertility remains unknown. Therefore, in this study, we first investigated the expression patterns of GGCX and MGP in the mouse epididymis. Subsequently, we generated a *Ggcx^K325Q^* knock-in mouse model that corresponded to the SNP mutation of GGCX in humans via the CRISPR-Cas9 technology to further examine the effect of the rs699664 mutation of GGCX on mouse fertility and epididymal calcium homeostasis. The results showed that calcium homeostasis of the epididymis was not disturbed after *Ggcx^K325Q^* mutation, and that the *Ggcx^K325Q−/−^* mice were fertile, indicating that the *Ggcx^K325Q^* mutation does not affect male fertility in mice.

## 2. Materials and Methods

### 2.1. Animals

Mice were obtained from the Laboratory Animal Center of Air Force Medical University and were housed in a room with a constant humidity and a temperature of 23 ± 2 °C. They were provided unlimited access to food and water. All the 2-month-old male mice were sacrificed by cervical dislocation before tissue collection. All the experiments and studies on laboratory animals were carried out according to the guidelines approved by the Ethics Committee for Animal Care and Experiments of the Air Force Medical University (project identification code 20210343).

### 2.2. Generation of Ggcx^K325Q−/−^ Mice via CRISPR/Cas9 Technology

*Ggcx^K325Q−/−^* knock-in mice were generated using CRISPR/Cas9-mediated genome engineering. The gRNA (GCAGCTCTTGCAGCCTTTTCGGG) to the mouse *Ggcx* gene, the donor oligo containing p.K325Q (AAA to CAA) mutation, and Cas9 were co-injected into fertilized mouse eggs to generate targeted knock-in offspring. F0 founder animals were identified by PCR followed by sequence analysis, and they were bred into wild-type mice to test germline transmission and F1 animal generation. The PCR primers were as follows: forward, 5′-CTTCTAGGTTCGGGAAGCAGTTC-3′ and reverse, 5′-CAGCATTGGTTTAGTGCTTCTCTAC-3, yielding an 873 bp fragment.

### 2.3. Fertility Assay

To investigate the fertility of the *Ggcx^K325Q−/−^* mice, 2-month-old wild-type and *Ggcx^K325Q−/−^* male mice (*n* = 5) were caged with two 2-month-old wild-type C57BL/6J females. The vaginal plugs were checked the next day, and the male mice were separated for the next two days to rest. After that, the male mice were caged with two new 2-month-old female mice. Females with positive vaginal plugs were housed separately. The average litter size of each mouse line was calculated and recorded.

### 2.4. Total RNA Extraction and RT-qPCR

Total RNA was extracted from 2-month-old wild-type mouse epididymides using TRIzol reagent (Takara, Shiga, Japan), following the manufacturer’s instructions. RNA was reversely transcribed into cDNA using a Primer Script RT reagent kit (RR047A; Takara, Shiga, Japan). The cDNA was diluted for RT-quantitative PCR (RT-qPCR) with SYBR Green Master Mix (A25742, Thermo Scientific, Waltham, MA, USA). The primer sequences for rat GGCX and MGP were designed as follows: GGCX: 5′-TGTCGTGACCCTGCTTAACAAACC-3′, 5′-GCACACATCCAGCCCATCCAAG-3′; MGP: 5′-CCCTGTGCTACGAATCTCACGAAAG-3′; 5′-GGCTTGTTGCGTTCCTGGACTC-3′. The PCR protocol was 40 cycles of 95 °C for 15 s, 56 °C for 15 s, and 72 °C for 30 s. Each sample was analyzed in triplicate, and the experiment was repeated independently three times with similar results. The amplification of 18S was used as an internal control.

### 2.5. Western Blotting

Epididymis and kidney tissues were collected from 2-month-old wild-type mice, lysed in cold-RIPA buffer with the addition of a protein inhibitor cocktail (04693132001, Roche, Basel, Switzerland) for 30 min on ice, and centrifuged at 12,000 rpm for 15 min at 4 °C. The protein concentration was determined using a bicinchoninic acid (BCA) protein assay (MI00606A, Mishushengwu, Xi’an, China). Equal amounts of protein samples were subjected to 12.5% SDS-PAGE and transferred to nitrocellulose membranes. The primary antibodies used were as follows: anti-GGCX (DF12616, Affinity, Cincinnati, OH, USA; 1:2000), anti-MGP (103734-1-AP, Proteintech, Chicago, IL, USA; 1:2000), and anti-β-actin (AF7018, Affinity, Cincinnati, OH, USA; 1:10,000). The secondary antibodies used were as follows: HRP-conjugated Affinipure Goat Anti-Rabbit IgG (H + L) (SA00001-2, Proteintech, Chicago, IL, USA; 1:10,000), HRP-conjugated Affinipure Goat Anti-Mouse IgG (H + L) (SA00001-1, Proteintech, Chicago, IL, USA; 1:10,000).

### 2.6. Histological Analysis

Epididymides from the 2-month-old wild-type and *Ggcx^K325Q−/−^* mice were removed and fixed in Bouin’s solution or 4% (*m*/*v*) paraformaldehyde (PFA) for 24 h. Subsequently, the tissues were dehydrated with 70, 80, 90, 95, and 100% ethanol, cleared with xylene, embedded in paraffin, and cut into 5 μm sections. After deparaffinization, tissue sections were stained with hematoxylin and eosin (H&E). For sperm preparation and staining, the cauda epididymis from the 2-month-old wild-type and *Ggcx^K325Q−/−^* mice were finely cut, immersed in PBS, and then incubated at 37 °C for 15 min to allow the spermatozoa to disperse throughout the PBS. After incubation, the sperms were spread on clean glass slides, fixed in 4% PFA for 15 min, and stained with H&E.

### 2.7. Immunofluorescence and TUNEL

For immunofluorescence (IF), mouse tissue slides underwent deparaffinization, rehydration, and antigen retrieval (boiled for 20 min in 0.01 M sodium citrate buffer). Then, tissues were blocked with 5% BSA (bovine serum albumin) for 1 h at room temperature. After blocking, the tissue slides were incubated overnight with primary antibodies at 4 °C. For sperm IF, after being fixed in 4% PFA for 15 min, sperm were permeabilized with 0.5% TritionX-100 for 10 min and blocked in 5% BSA for 1 h at room temperature. Then, the sperm slides were incubated with the primary antibody at 4 °C overnight. The primary antibodies were as follows: GGCX (16209-1-AP, Proteintech, Chicago, IL, USA; 1:50); MGP (PA68762, AntiProtech, Burlington, VT, USA; 1:100); and Ac-tubulin (ab24610, Abcam, Cambridge, UK; 1:200). The secondary antibody was as follows: FITC-conjugated Donkey anti-mouse (715-545-150, Jackson ImmunoResearch, West Grove, PA, USA; 1:400). The double labeling of two rabbit-origin primary antibodies was performed as previously described using a TSA amplification kit (G1235-100T; Servicebio, Wuhan, China) [28]. For the TUNEL assay, after blocking, the enzyme and labeling solutions were combined to form the reaction mixture, following the manufacturer’s protocol (11684795910, Roche, Basel, Switzerland). The mixture was then used to incubate the sections at 37 °C for 1.5 h. Photomicrographs were taken with a FV1000 confocal microscope (Olympus, Tokyo, Japan) or an Axio imager M1 fluorescence microscope (Zeiss, Oberkochen, Germany).

### 2.8. Computer-Assisted Sperm Analysis (CASA)

Spermatozoa from the 2-month-old wild-type or *Ggcx^K325Q−/−^* mice were collected from the cauda epididymis. The epididymal spermatozoa were extruded and suspended in HTF (human tubal fluid) culture medium, followed by a 20 min incubation at 37 °C, and then analyzed using the CASA system (SAS Medical, Beijing, China).

### 2.9. Ca^2+^ Measurement Assay

The epididymal Ca^2+^ density was detected by a calcium colorimetric assay kit (Beyotime, Chengdu, China). In brief, the epididymal epithelium tissues were homogenized in lysates and centrifuged to obtain 50 μL of the supernatant. Then, 75 μL Chromogenic Reagent and 75 μL Calcium Assay Buffer were added to 50 μL of tissue lysates and epididymal fluid, which were extracted using the method described previously [29], followed by a 10 min incubation at 37 °C. Finally, a Multiskan Spectrum (Bio-Tek, Winooski, VT, USA) was used to measure the absorbance at 575 nm.

### 2.10. Phylogenetic Analyses

Multiple alignments of amino acid sequences were downloaded from the Uniprot database (https://www.uniprot.org/, accessed on 10 January 2024), and phylogenetic trees were constructed using the MEGA 7 software with the Maximum Parsimony method.

### 2.11. Statistical Analysis

The statistical analysis was conducted with GraphPad PRISM 9.3.1 software, and the results were expressed as the mean ± SEM. All the experiments were independently repeated at least three times. Statistical significance was examined using the two-tailed unpaired Student’s *t* test. The significance of the data is presented as *p* > 0.05 (ns), *p* < 0.05 (*), 0.01 (**), 0.001 (***), and 0.0001 (****).

## 3. Results

### 3.1. Expression Patterns of GGCX and MGP in Mouse Epididymis

**Figure 1:**
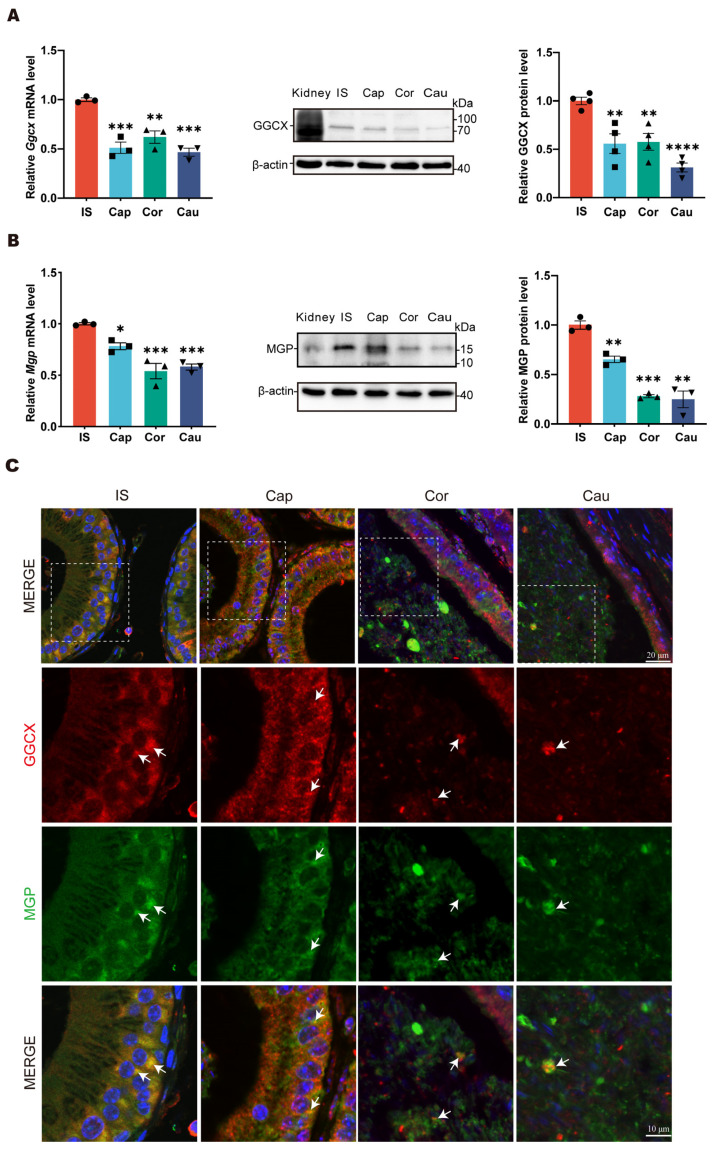
The expression patterns of GGCX and MGP in adult mouse epididymis. (**A**) Expression pattern analysis of GGCX in different regions of the epididymis, using RT-qPCR (*n* = 3) and Western blotting (*n* = 5). The representative Western blotting gels and statistical results are presented. Data are presented as mean ± SEM. * *p* < 0.001, ** *p* < 0.001, *** *p* < 0.001, **** *p* < 0.0001. IS, initial segment; Cap, caput; Cor, corpus Cau, cauda. (Kidney: positive control). (**B**) Expression pattern analysis of MGP in different regions of the epididymis, using RT-qPCR (*n* = 3) and Western blotting (*n* = 4). The representative Western blotting gels and statistical results are presented (Kidney: positive control). Data are presented as mean ± SEM. * *p* < 0.001, ** *p* < 0.001, *** *p* < 0.001. (**C**) Double immunofluorescence labeling of GGCX (red) and MGP (green) in different regions of the epididymis; the nuclei were stained with DAPI (blue). Dashed boxes show the localization of the enlarged images. Colocalization signals (yellow) are indicated with white arrows.

To gain insights into the GGCX and MGP expression patterns, we performed RT-qPCR and Western blotting analyses of GGCX and MGP in the initial segment, caput, corpus, and cauda regions of the mouse epididymis. A kidney from an adult mouse was used as a positive control for Western blotting. The results showed that the expression level of GGCX was highest in the initial segment of epididymis, while it was relatively weak in the caput, corpus, and cauda regions (Figure 1A). Similar to GGCX, MGP was also expressed in all segments of the mouse epididymis and displayed the highest expression level in the initial segment (Figure 1B). Immunofluorescence was performed to further examine the colocalization of GGCX and MGP in the mouse epididymis. In the initial segment, we could observe that GGCX and MGP were mainly colocalized in the basal compartment of the initial segment, while in the corpus and cauda regions, the colocalization signals were mainly observed in the lumen (Figure 1C). The gradual accumulation of MGP-positive signals was observed in the apical compartment of epithelial cells along the epididymis, particularly in the corpus region. These data suggest that in situ MGP carboxylation by GGCX occurs not only in epididymal epithelial cells but also in the epididymal luminal microenvironment of the mouse epididymis.

### 3.2. Generation of Ggcx^K325Q^ Knock-in Mice

**Figure 2:**
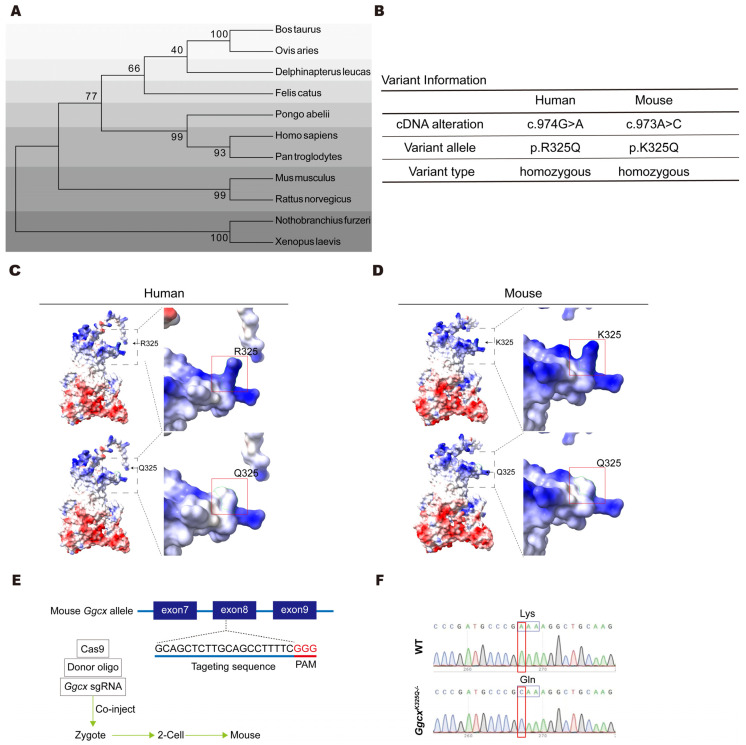
The construction of the GGCX 325 site and the generation of *Ggcx^K325Q−/−^* mice. (**A**) Phylogenetic trees of GGCX homologous proteins in mammalian species. The numbers in the dendrogram represent bootstrap values (%). (**B**) The variant information of GGCX between humans and mice. (**C**,**D**) Poisson–Boltzmann electrostatic maps of human (**C**) and mouse (**D**) normal GGCX and the Q325 variant show changes to the charge (arrow) of the protein. Dashed boxes show the localization of the enlarged images. (**E**) Strategy schematic of *Ggcx^K325Q−/−^* mice construction using CRISPR-Cas9-mediated genome editing. (**F**) Sanger sequencing results of knock-in mice confirmed the successful mutation from K325 to Q325 in the *Ggcx* gene.

The phylogenetic tree of GGCX shows that GGCX is a relatively conserved protein in different species across evolution (Figure 2A). In humans, the SNP rs699664 manifested as p.R325Q (CGA to CAA), whereas in mice, it presented as p.K325Q (AAA to CAA) (Figure 2B). Although this site is not entirely conserved, the analysis of the Poisson–Boltzmann electrostatic maps model (generated by ChimeraX) indicates that both p.R325Q and p.K325Q exhibited the same changes in charge after mutation (Figure 2C,D). The R325 site of GGCX is located in close proximity to the MGP binding domain (amino acids 343–355). The mutation of a positively charged moiety to a neutral one at this site may create an unfavorable condition for MGP binding during carboxylation, resulting in decreased MGP carboxylation and thereby to an increased calcium mineralization and Ca^2+^-mediated proliferation of stress granules. Eventually, a disordered epididymal luminal microenvironment is created, causing impaired sperm maturation and male infertility. To investigate the impact of this mutation on calcium balance in the epididymis, we utilized CRISPR/Cas9 technology to create *Ggcx^K325Q^* knock-in mice (Figure 2E). The Sanger test showed that we successfully established a mouse with *Ggcx* 973A>C, resulting in a lysine-to-glutamine missense mutation (Figure 2F).

### 3.3. Ggcx^K325Q^ Does Not Affect the Normal Growth and Fertility of Mice

**Figure 3:**
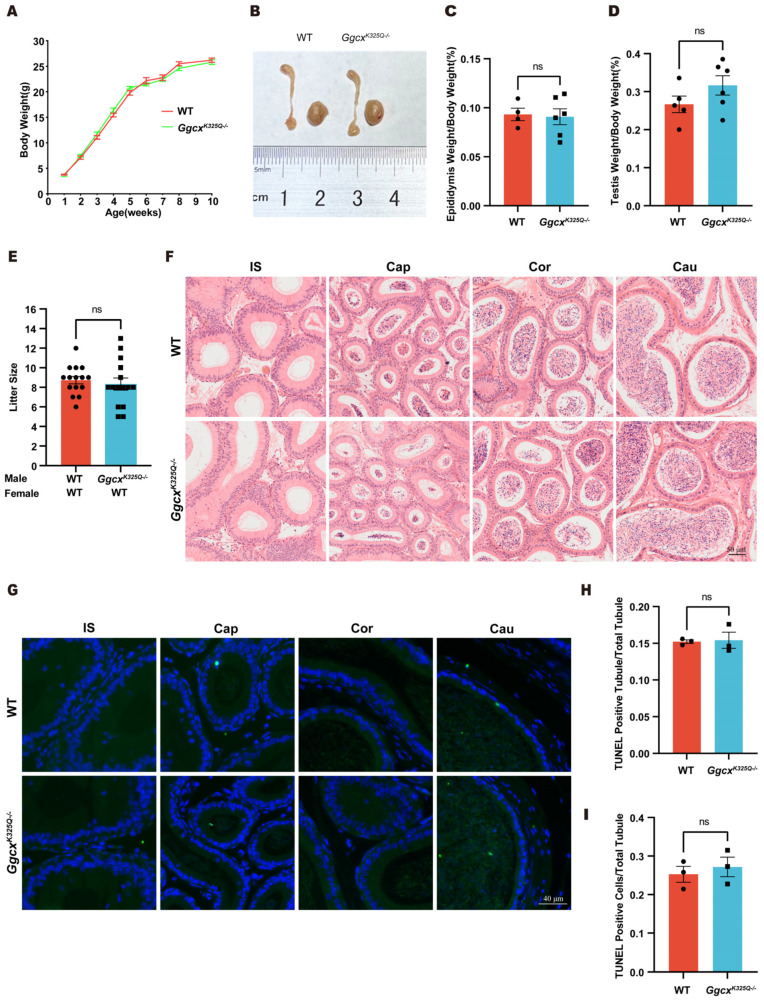
*Ggcx^K325Q−/−^*mice show normal development and fertility. (**A**) Body weight of wild-type mice and *Ggcx^K325Q−/−^* mice from 1 to 10 weeks of age. (*n* = 3). Data are presented as mean ± SEM. (**B**) Representative images of adult wild-type and *Ggcx^K325Q−/−^* testis and epididymis. (**C**) Epididymis weight/body weight ratios in adult wild-type (*n* = 4) and *Ggcx^K325Q−/−^* mice (*n* = 6). Data are presented as mean ± SEM. ns *p* > 0.05. (**D**) Testis weight/body weight ratios in adult wild-type (*n* = 5) and *Ggcx^K325Q−/−^* mice (*n* = 6). Data are presented as mean ± SEM. ns *p* > 0.05. (**E**) Average litter size of wild-type and *Ggcx^K325Q−/−^* mice. (*n* = 3 independent experiments). Data are presented as mean ± SEM. ns *p* > 0.05. (**F**) H&E staining of epididymides from adult wild-type and *Ggcx^K325Q−/−^* mice. (**G**–**I**) Different regions of wild-type and *Ggcx^K325Q−/−^* mice epididymis were stained for the TUNEL (green) probe, and the nuclei were stained with DAPI (blue) (**G**). Average TUNEL positive apoptotic tubule counts (**H**). Average TUNEL positive apoptotic cell counts (**I**) (*n* = 3). Data are presented as mean ± SEM. ns *p* > 0.05.

*The Ggcx^K325Q−/−^* mice displayed normal development. The body, testis, and epididymis weights did not significantly differ between the *Ggcx^K325Q−/−^* and wild-type mice (Figure 3A–D). To investigate whether the *Ggcx^K325Q−/−^* mutation affects male fertility, we performed mating tests and found that the *Ggcx^K325Q−/−^* mice were fertile, with their litter sizes similar to those of wild-type mice (Figure 3E). To examine whether any subtle epididymal defects existed in epididymis of the *Ggcx^K325Q−/−^* mice, we carried out H&E staining of the epididymis. The results revealed that the epididymides of the *Ggcx^K325Q−/−^* mice displayed normal structures and no obvious defects (Figure 3F). Moreover, we did not detect any abnormally increased number of epididymal apoptotic cells in the *Ggcx^K325Q−/−^* mice compared with the wild-type mice using the TUNEL test, and the statistical results also did not show any significant difference between the *Ggcx^K325Q−/−^* mice and the control group (Figure 3G–I). Thus, we concluded that the *Ggcx^K325Q^* mutation does not affect normal growth, male fertility, or epididymal cell apoptosis in mice.

### 3.4. Ggcx^K325Q^ Does Not Affect Sperm Counts, Morphology, and Motility

**Figure 4:**
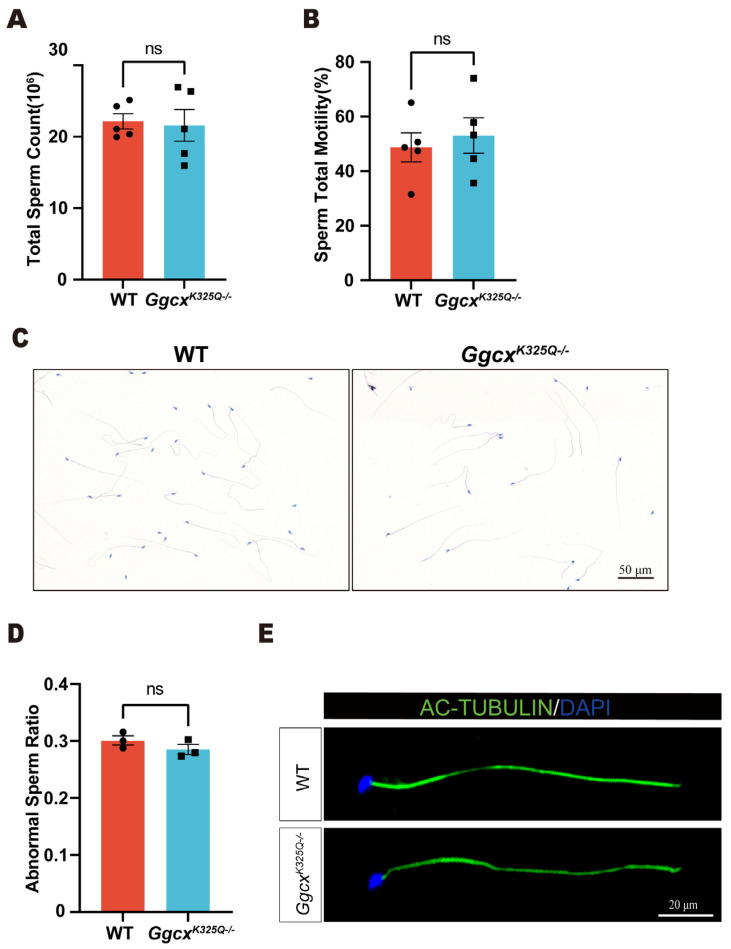
Spermatozoa appear normal in *Ggcx^K325Q−/−^* mice. (**A**) Sperm count from adult wild-type and *Ggcx^K325Q−/−^* mice. (*n* = 5). Data are presented as mean ± SEM. ns *p* > 0.05 (**B**) Sperm total motility from adult wild-type and *Ggcx^K325Q−/−^* mice. (*n* = 5). Data are presented as mean ± SEM. ns *p* > 0.05 (**C**) Histological analysis of the spermatozoa of adult wild-type and *Ggcx^K325Q−/−^* mice, using H&E staining. (**D**) Average abnormal sperm ratio in adult wild-type and *Ggcx^K325Q−/−^* mice. (*n* = 3). Data are presented as mean ± SEM. ns *p* > 0.05 (**E**) Immunofluorescence staining of Ac-tubulin (green) in wild-type and *Ggcx^K325Q−/−^* mouse spermatozoa. Sperm heads were stained with DAPI (blue).

Morphologically and functionally normal sperm are essential for fertilization. Computer-assisted sperm analysis (CASA) was used to evaluate the semen quality of the *Ggcx^K325Q−/−^* mice. We found that there was no significant change in the sperm amount and sperm motility ratio in the *Ggcx^K325Q−/−^* mice compared to the wild-type mice (Figure 4A,B). H&E staining of the sperm showed no obvious changes in sperm morphology (Figure 4C,D). In addition, IF staining of the sperm flagella marker Ac-tubulin showed that the sperm flagellum was intact in the *Ggcx^K325Q−/−^* mice (Figure 4E). In summary, the *Ggcx^K325Q−/−^* mice exhibited no significant abnormalities in sperm morphology, sperm count, or sperm motility.

### 3.5. Calcium Homeostasis in Epididymal Luminal Is Normal in Ggcx^K325Q−/−^ Mice

**Figure 5:**
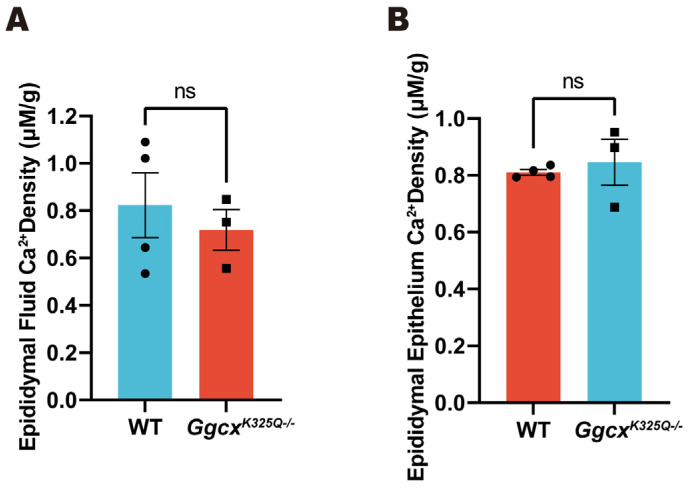
Ca^2+^ levels in the epididymal fluid and epithelium are normal in *Ggcx^K325Q−/−^* mice. (**A**) Epididymal fluid Ca^2+^ density of the adult wild-type and *Ggcx^K325Q−/−^* mice. (*n* = 4). Data are presented as mean ± SEM. ns *p* > 0.05 (**B**) Epididymal epithelium Ca^2+^ density of the adult wild-type and *Ggcx^K325Q−/−^* mice. (*n* = 4). Data are presented as mean ± SEM. ns *p* > 0.05.

A low Ca^2+^ concentration in the epididymal lumen is essential for sperm maturation and storage. Therefore, the total epididymal fluid Ca^2+^ concentration and epididymal epithelial Ca^2+^ concentration were measured in the *Ggcx^K325Q−/−^* mice. The results indicate that there were no significant changes in Ca^2+^ concentration in the epididymal fluid and epididymal epithelium of the *Ggcx^K325Q−/−^* mice compared with the wild-type mice (Figure 5).

## 4. Discussion

Vitamin K2-related GGCX-dependent carboxylation of MGP is crucial for maintaining calcium homeostasis in the epididymis. The significantly increased frequency of the rs699664 mutation in the *GGCX* gene among AZS patients suggests that this mutation may leads to AZS. In the present study, we investigated GGCX and MGP expression patterns in the mouse epididymis and generated a *Ggcx^K325Q^* knock-in mouse model to explore the impact of this mutation on epididymal calcium homeostasis and male fertility. Unexpectedly, the *Ggcx^K325Q^^−/^^−^* mice exhibited normal development and fertility.

Epididymal Ca^2+^ homeostasis is essential for sperm activity and male fertility [8], and calcium homeostasis maintenance is regulated by a variety of molecules. Shum et al. systematically described molecules that are crucial in maintaining epididymal calcium homeostasis, including the calcium-selective channel TRPV6, calcium extrusion pump PMCA4, and the GGCX-MGP system [8]. As an important calcium homeostasis maintenance molecule, MGP requires vitamin K-dependent gamma-carboxylation [30], and GGCX is the only gamma-carboxylation enzyme in the cells and has no relevant homology to any known enzyme families [8]. Our results show that both GGCX and MGP were highly expressed in the initial segment of the mouse epididymis and colocalized in the epithelial cells of the initial segment and caput region, as well as in the lumen of the corpus and cauda regions of the mouse epididymis. However, GGCX did not always colocalize with MGP in the mouse epididymis, which is consistent with our previous study in rats [12]. This is most likely because GGCX, as the carboxylase of MGP, only temporarily binds to its substrate. Moreover, GGCX and MGP were highly expressed in the corpus and cauda regions of epididymis but were scarcely observed in the initial segment of epididymis in the rat [12], which differed from the expression patterns in mice. We speculate that the different expression patterns in the mouse and rat epididymis are caused by species differences. A large number of studies have demonstrated that MGP plays an indispensable role in calcium regulation, especially in organs or tissues where it is highly expressed, such as bone and blood vessels [9,31,32,33]. Similarly, MGP was highly expressed in the initial segment of the mouse epididymis and colocalized with GGCX in this region. Thus, we speculate that the GGCX-MGP system may participate in Ca^2+^ regulation in the initial segment of the mouse epididymis, which merits further investigation.

The rs699664 mutation in the *GGCX* gene has been associated with AZS in humans [12]. To further investigate the effect of the *Ggcx^K325Q^* mutation, which corresponds to the rs699664 mutation of *GGCX* in humans, on mouse fertility and epididymal calcium homeostasis, *Ggcx^K325Q^^−/^^−^* mice were generated and examined under normal conditions in this study. Unexpectedly, the *Ggcx^K325Q^^−/^^−^* mice exhibited normal sperm morphology, sperm count, sperm motility, and epididymal morphology compared with the wild-type mice, and a TUNEL analysis showed no significant apoptosis in *Ggcx^K325Q^^−/^^−^* epididymal cells. As an aberrantly high Ca^2+^ level in the epididymis could result in defective spermatozoa, impaired fertility, and cell apoptosis [12,34,35,36], we further verified Ca^2+^ levels in the epididymis of the *Ggcx^K325Q^^−/^^−^* mice, but it did not differ significantly from that in wild-type mice, so the normal sperm and epididymal morphology of the *Ggcx^K325Q^^−/^^−^* mice may be related to unimpaired calcium homeostasis to some degree. In addition, we observed no significant differences in body weight, testis/body weight ratio, epididymis/body weight ratio, and the number and size of litters between the *Ggcx^K325Q^^−/^^−^* mice and wild-type mice, indicating that the *Ggcx^K325Q^* mutation does not affect male development and fertility.

Over the past decades, many mutated genes derived from infertile patients have been verified in gene knockout or mutant animal models [37]. In this study, male *Ggcx^K325Q^^−/^^−^* mice exhibited normal fertility, which is contradictory to the previous associations of rs699664 with AZS in the Chinese population [12]. We speculate that there are two possible reasons for this difference. First, discrepancies between human and mouse models may account for the different phenotypes. In the present study, the *Ggcx^K325Q^^−/^^−^* mice exhibited normal epididymal Ca^2+^ concentrations, suggesting that this mutation did not affect the carboxylation levels of MGP. However, a recent study by Hao et al. indicated a decrease in MGP carboxylation levels in HEK293 cells with this mutation, which differed from our mouse model results [38]. Studies of DNAH1 have also revealed differences between humans and mice. Sperm from *DNAH1*-mutant (c.117881G>A) patients has shown defects in the sperm flagellar axonemal structure [39], while Dnah1-deficient mice showed no such anomalies [40]. Primate genome editing models may be more suitable for investigating potential disease-causing mutations found in humans due to the higher similarities between primates and humans [41]. Second, the previous sample size may not reflect the actual situation. In our previous study, 29 of 199 (14.6%) AZS patients and 8 of 110 (7.3%) normal individuals carried the rs699664 mutation [12]. This difference was statistically significant after performing Fisher’s exact test (*p* = 0.041). However, the mutation carrier rate of normal individuals in our study (7.3%) was lower than that of GnomAD East Asian males (10.2%). While we compared the mutation carrier rate of AZS patients with that reported by GnomAD using Fisher’s exact test, no significant difference was found (*p* = 0.146). This difference indicates that the results may be biased due to the insufficient sample size of normal individuals in our previous study.

In summary, we explored the GGCX and MGP expression patterns in the mouse epididymis and confirmed that the mouse *Ggcx^K325Q^* mutation does not affect male fertility in mice. The present study provided some insights for further exploring epididymal calcium homeostasis and male infertility.

## Data Availability

The original contributions presented in the study are included in the article, further inquiries can be directed to the corresponding author.
